# Toxic Epidermal Necrolysis and Steven–Johnson Syndrome During the Postpartum Period: A Literature Review with a Rare Case Presentation

**DOI:** 10.3390/jcm15010017

**Published:** 2025-12-19

**Authors:** Natalia Katarzyna Mazur-Ejankowska, Maciej Ejankowski, Magdalena Emilia Grzybowska, Jakub Żółkiewicz, Ewa Gostkowska, Wioletta Barańska-Rybak, Dariusz Grzegorz Wydra

**Affiliations:** 1 Department of Gynecology, Obstetrics and Neonatology, Medical University of Gdansk, 80-210 Gdansk, Poland; 2 Clinic of Obstetrics and Gynecology, Gynecological Oncology and Endocrine Gynecology, University Clinical Centre, 80-952 Gdansk, Poland; 3 First Doctoral School, Medical University of Gdansk, 80-210 Gdansk, Poland; 4 Department of Dermatology, Venereology and Allergology, Medical University of Gdansk, 80-210 Gdansk, Poland; 5 Clinic of Dermatology, Venereology and Allergology, University Clinical Centre, 80-952 Gdansk, Poland; 6 Gynecological and Obstetrical Practice, 80-244 Gdansk, Poland

**Keywords:** Toxic Epidermal Necrolysis (TEN), Steven–Johnson Syndrome (SJS), Lyell’s syndrome, postpartum, obstetrics, polypharmacy, complication

## Abstract

**Introduction:** Toxic Epidermal Necrolysis (TEN) and Steven–Johnson Syndrome (SJS) are rare yet dangerous dermatological emergencies presenting as necrosis of the skin and mucous membranes due to an immune reaction which may be associated with the use of pharmaceuticals—predominantly non-steroidal anti-inflammatory drugs (NSAIDs), antibiotics, and antiretroviral drugs. During the postpartum period, women are administered numerous pharmaceuticals, including NSAIDs, analgesics, and antibiotics, due to pain and their susceptibility to infections, exposing them to potential adverse effects including allergies and immune reactions. **Case Report and Review:** The case reported here is a rare description of a patient in the early postpartum phase who presented with epidermal necrolysis whilst remaining hospitalized after a cesarean delivery. The multidisciplinary approach, early diagnosis, and treatment ensured the patient’s full recovery. Intravenous immunoglobulin treatment resulted in a rapid therapeutic effect. This literature review offers an insight into the epidemiology, diagnostic process, and treatment of this infrequent dermatological syndrome during the postpartum period. **Results:** Antibiotic treatment is a common culprit of TEN in this population; hence, clinicians should remain vigilant during antibiotic administration. Differential diagnosis with toxic shock syndrome is crucial, as TEN and SJS symptoms may mimic sepsis, which is a more common diagnosis in the postpartum period. **Conclusions:** The condition of the skin during the postpartum period should be closely monitored, as some systemic diseases may manifest abruptly as profound, postpartum hormonal changes affect the immunological response. Upon the discovery of suspicious skin lesions concomitant with systemic symptoms, an immediate multidisciplinary approach involving obstetricians and dermatologists is key to a rapid diagnosis and treatment to avoid maternal mortality.

## 1. Introduction

Toxic Epidermal Necrolysis (TEN) is a severe drug-induced febrile mucocutaneous reaction. TEN, Steven–Johnson Syndrome (SJS), and SJS/TEN overlap syndrome are considered to form a spectrum of one disease with a varying severity [1]. TEN causes epidermal detachment of more than 30% of the body surface area (BSA). SJS involves under 10% of the BSA and SJS/TEN overlap is diagnosed in intermediate cases [2]. Antibiotics, non-steroidal anti-inflammatory drugs (NSAIDs), antiretroviral drugs, and anticonvulsants, as well as bacterial and viral infections, may trigger the onset of TEN, SJS, and TEN/SJS overlap syndrome [3].

This paper describes a clinically difficult case of SJS in a patient during the first few days of the postpartum period after a cesarean delivery. Rapid treatment, including intravenous immunoglobulin (IVIG) administration, was initiated in consultation with the dermatology consultants. Careful management of the patient’s exacerbating symptoms and systemic treatment led to a stable improvement.

The fragile time of the postpartum period, especially after cesarean delivery, requires frequent NSAID administration and, additionally, due to increased susceptibility to infections, antibiotics are often used empirically. In the postpartum period. women are given multiple pharmaceuticals to deal with pain and infections, such as endometritis or wound infection, exposing them to the potential side effects of newly administered pharmaceuticals [4,5]. Childbirth plays a crucial role in the immunomodulation of the mother, as immunity deceases due to elevated levels of pro-inflammatory factors that may increase the risk of SJS and TEN.

Additionally, this paper offers a narrative literature review, including epidemiology and treatment of TEN and SJS during the pregnancy and postpartum periods, which may be helpful to obstetricians, dermatologists, and other medical professionals. Insights into differential diagnosis and multimodal treatment methods are discussed and evaluated with regard to the postpartum population.

## 2. Case Report

A 29-year-old primipara was admitted to a tertiary hospital because of an abnormal cardiotocography (CTG) conducted in the 36th week of an uncomplicated-to-date pregnancy. Following the hospital admission, the amniotic membranes spontaneously ruptured, causing leakage of clear amniotic fluid, and the patient was qualified for pharmacological induction of labor with oxytocin. In the course of labor, the patient was administered a standard course of empirical antibiotics: ampicillin (1 g every 4 h) as prophylaxis of intrauterine infection due to Group B streptococcus (GBS) vaginal colonization. Finally, due to prolonged decelerations of the fetal heart rate, the patient qualified for an immediate cesarean section under subarachnoid analgesia.

In the early postpartum period, ampicillin was continued in the standard endometritis prophylaxis protocol due to a high risk of systemic infection and elevation of C-reactive protein (CRP) levels. In a bacterial cervical swab, which was taken at admission, *Ureaplasma urealyticum* was cultured and azithromycin treatment (500 milligrams daily, orally for 5 days) was administered. Concomitantly, the patient received the standard postoperative pain relief treatment, including oral acetaminophen (1 g) and ibuprofen (400 milligrams) alternately, every 6 h.

On day 3, after the cesarean section, which was day 4 of ampicillin administration, day 3 of acetaminophen and ibuprofen administration, and day 2 of azithromycin administration, a widespread eruption of 1- to 2-mm red lesions appeared on the patient’s abdomen and limbs. Initially, an allergic reaction to the administered antibiotics was suspected, as the reaction occurred within 20 minutes of intravenous ampicillin administration. A daily analysis of the patient’s condition, medications, dosage, laboratory results, and medical interventions is presented in Table 1.

Due to the suspected drug-induced reaction, a decision was made to discontinue both ampicillin and ibuprofen. Upon patient’s examination by the on-call obstetrician, cetirizine, topical fluticasone, and emollients were recommended. The following day, a severe exacerbation of the cutaneous lesions occurred, and a dermatological consultation was ordered. Based on the clinical presentation, the consulting dermatologist suspected a morbilliform drug eruption and recommended the introduction of oral prednisone at 20 milligrams daily. Despite the initiation of systemic glucocorticosteroid therapy, on the following day, the lesions transformed into purpura with bullae containing serous fluid. The patient’s body temperature reached 39 degrees Celsius. Immediately, the patient received 200 milligrams of hydrocortisone intravenously, which had no effect, and another urgent dermatological consultation was requested. A suspicion of an early sepsis was considered, the patient’s blood samples were sent for bacterial culture and sensitivity testing and procalcitonin levels where requested, which remained within the normal range.

Physical examination revealed extensive and rapidly progressive mucocutaneous involvement. The dorsal aspects of the feet were violaceous, while the soles and palms showed multiple, well-demarcated erythematous macules. Confluent purpuric patches were present on the groins. Numerous superficial erosions were observed on the labia majora and minora and perineal skin, accompanied by epidermal detachment with a positive Nikolsky sign in this region. On the back, violaceous discoloration with multiple small (1–2 mm) pustules was observed. On the chest and abdomen, confluent erythematous papules and pustules on an erythematous base were identified, and a cluster of well-tensed bullae were found on the right upper arm. Moreover, confluent erythematous maculopapular eruptions were present on the forearms. The facial skin was edematous, with scattered papular lesions and perioral and perinasal bullous eruptions with concomitant yellowish crusting. The vermilion border was spared. Marked edema and erythema of both upper and lower eyelids were observed, along with conjunctival hyperemia. In the oral cavity, superficial erosions affected the buccal mucosa.

**Figure 1:**
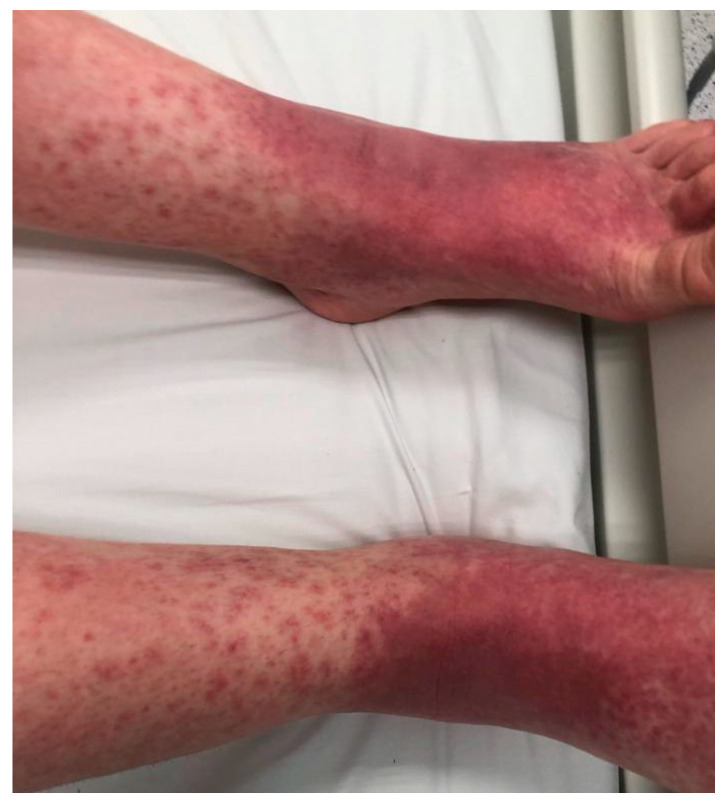
Patient’s lower limbs before intravenous immunoglobulins (IVIG) administration on day 3 after the initial reaction; six days after the cesarean delivery. On the dorsal aspect of the feet, confluent erythematous and purpuric macules were observed. Similar lesions were also observed on the palmar surfaces of the hands.

**Figure 2:**
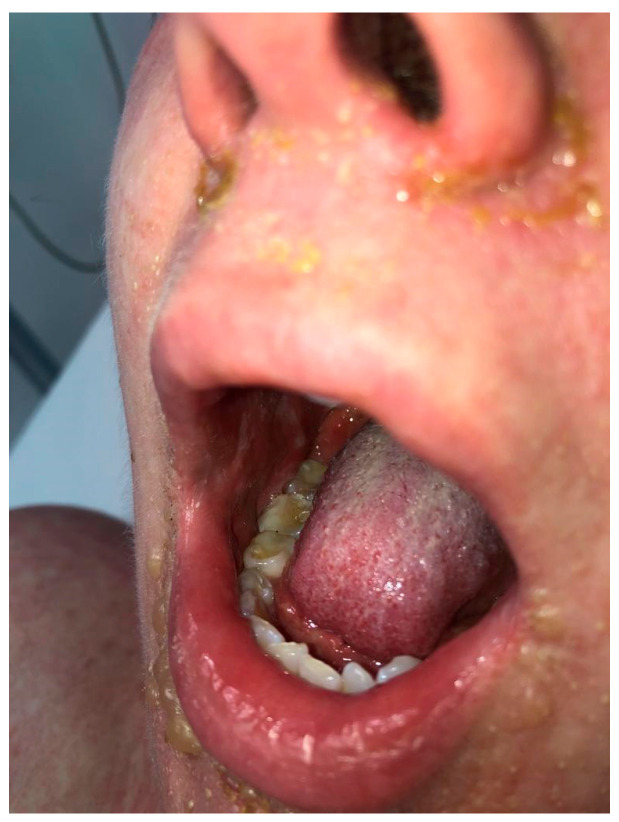
Patient’s face and oral mucosa before IVIG administration on day 3 after the initial reaction; six days after the cesarean delivery. Bullous lesions were observed around the nose and mouth, with features of impetiginization.

**Figure 3:**
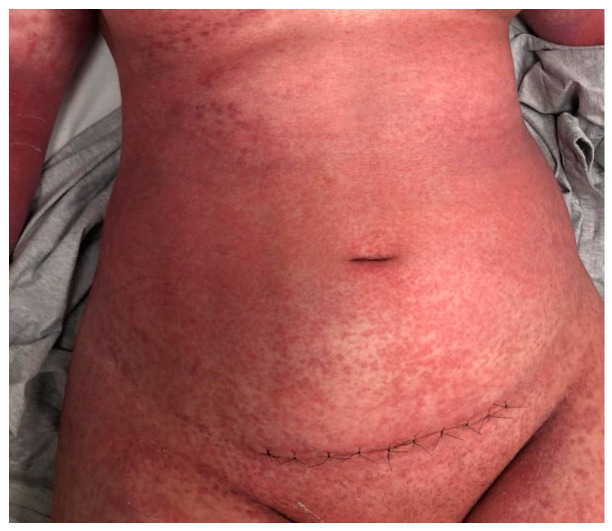
Patient’s chest and abdomen before IVIG administration on day 3 after the initial reaction; six days after the cesarean delivery. Confluent erythematous papules and pustules on an erythematous base were observed on the chest and abdomen.

Considering the rapid progression from extensive erythematous and dusky red macules to blister formation and epidermal detachment, the presence of mucosal erosions, conjunctival involvement, significant odynophagia, and the overall critical condition of the patient, a diagnostic biopsy was waived. A preliminary clinical diagnosis of TEN was established (Figure 1, Figure 2 and Figure 3) following a joint consultation with a board of professors in dermatology. The differential diagnosis pathway leading to establishment of the final diagnosis is summarized in Table 2.

Due to a further exacerbation of the patient’s symptoms, intravenous fluids were initiated, the patient’s bladder was catheterized, and diuresis was noted hourly with constant monitoring of life parameters, which, apart from one hypotensive and tachycardic event, remained stable. Upon a multidisciplinary case conference, the patient was referred to a specialist Burns and Plastic Surgery Unit; however, the transfer was impossible due to the unit being at a full capacity; hence, the treatment was continued in the tertiary center.

The patient’s blood, urine, lochia, breastmilk, swabs of skin lesions, and intravenous catheters were examined microbiologically; no bacterial infection or colonization were found. A chest X-ray was performed with no pathological findings. *Mycoplasma genitalium* and *Mycoplasma hominis* infection and colonization were also excluded.

**Figure 4:**
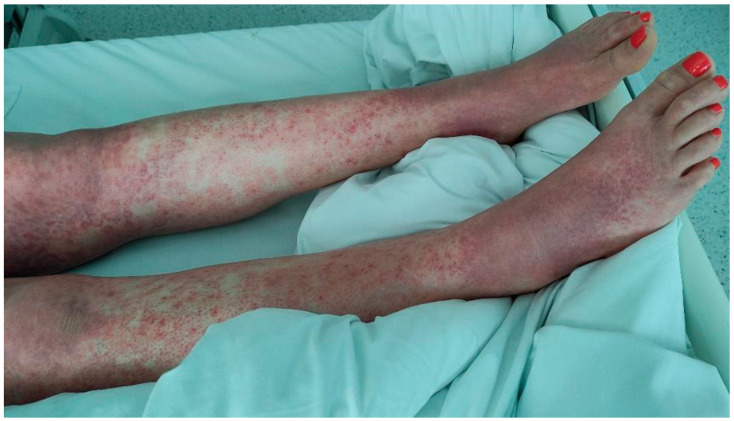
The patient’s lower limbs after the administration of the first dose of IVIG on day 5 after the initial reaction; eight days after the cesarean delivery. A partial improvement in the cutaneous lesions was observed.

**Figure 5:**
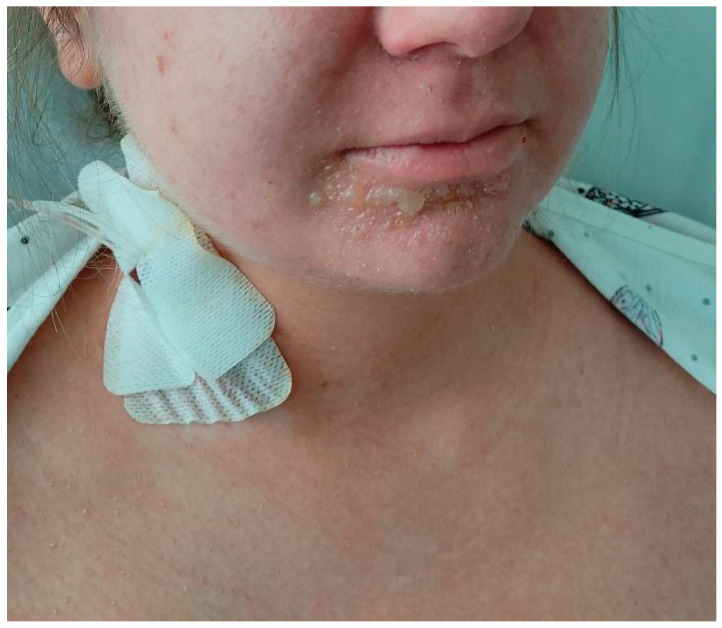
The patient’s face after the administration of the first dose of IVIG on day 5 after the initial reaction; eight days after the cesarean delivery. A partial improvement in the periorificial area was observed following the application of topical chloramphenicol ointment; however, perioral blistering persisted.

**Figure 6:**
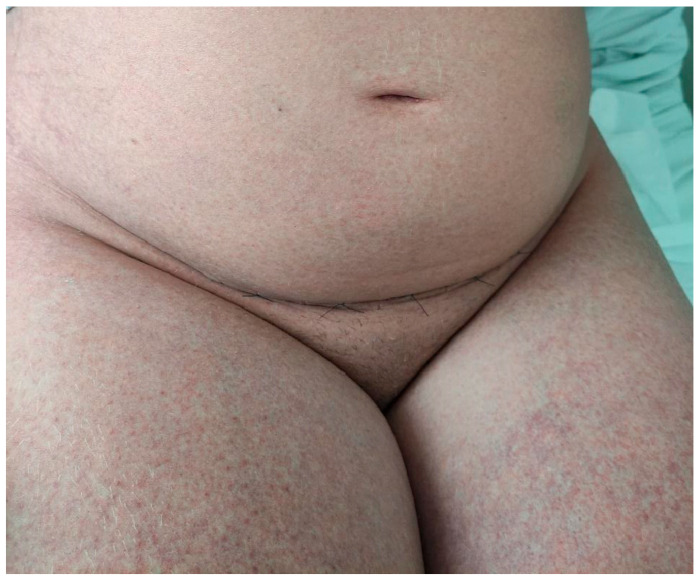
The patient’s abdomen after the administration of the first dose of IVIG on day 5 after the initial reaction; eight days after the cesarean delivery. A substantial improvement in the cutaneous involvement of the abdominal and thigh regions was observed.

A decision to pharmacologically cease lactation with bromocriptine was made due to the use of multiple drugs which are known to pass into the breastmilk and to avoid a further exacerbation of patient’s systemic symptoms. Based on a multidisciplinary expert consultation and supported by evidence from one of the larger retrospective studies [6], off-label treatment of intravenous immunoglobulins (IVIGs) was initiated at a dose of 1 mg/kg/day for 3 consecutive days. A rapid improvement in the patient’s condition was observed after the first day of IVIG treatment (Figure 4, Figure 5 and Figure 6). Subsequently, the patient was consulted by an ophthalmologist for a cornea examination and a topical treatment was recommended. An ear, nose, and throat specialist conducted an oral and laryngeal examination; the condition of the mucous membranes was confirmed to be healing. Additionally, throughout the course of the hospitalization, the patient was supported by a psychologist with a good therapeutic effect. The patient’s general and skin condition gradually improved during the subsequent days. Further hospitalization was prolonged because of neonatal reasons which were unrelated to the case. During this period, the patient required methyldopa (250 mg, 3 times daily) administration due to mild hypertension, which might have been attributed to the steroid intake.

Fifteen days after the initial hospital admission, the patient was discharged without any complications and with acceptable skin healing. It is crucial to underline that the patient did not report any allergies to medications or adverse effects from antibiotics taken previously. The Algorithm of Drug Causality for Epidermal Necrolysis (ALDEN) score, aiming to establish the culprit drug, is presented in Table 3. It should be noted that ampicillin, azithromycin, and ceftriaxone each had an ALDEN score of 3, which means all three antibiotics were a possible culprit. Additionally, ibuprofen had an ALDEN score of 2, meaning it was also a possible culprit. As the initial skin reaction took place 20 minutes after the intravenous ampicillin infusion, it is most probable that ampicillin was the culprit drug; however, this cannot be stated with certainty.

The SCORTEN scale (SCORe of Toxic Epidermal Necrosis) was calculated to determine the estimated mortality rate, which was calculated at 3.2% (Table 4). Another prognostic tool, the ABCD-10 score, which is used to predict the risk of in-hospital mortality of patients with SJS/TEN, was calculated for our patient and the risk was estimated at 2.3% (Table 4).

Upon a careful reconsideration, a decision to alter the preliminary diagnosis from TEN to SJS was made, as the necrotic skin lesions developed in less than 10% of the BSA. Initially, TEN was strongly suspected as over 90% of the BSA of patient’s skin and oral mucosa presented with erythematous papules and pustules on an erythematous base. The rapid progression of skin lesions into blisters and the patient’s poor general condition were also indicative of a TEN diagnosis. However, the epidermal detachment was finally observed in under 10% of the BSA, which is in line with an SJS diagnosis.

The multidisciplinary cooperation with the dermatology department enabled a proactive therapeutic approach and a rapid modification of the treatment upon inadequate improvement. The causative agent could not be confirmed due to polypharmacy; however, ampicillin was suspected.

## 3. Review

TEN or SJS diagnosis during the postpartum period is rare and the data regarding the treatment methods and differential diagnosis is limited. An extensive literature search revealed only two other cases of TEN during the postpartum period. No cases of SJS during the postpartum period were found.

The first case discusses a presentation of TEN on the fourth post-cesarean section (CS) day, which was performed due to cephalopelvic disproportion, in a 28-year-old woman with no concomitant diseases [7]. The differential diagnosis included a septic shock syndrome diagnosis due to rapidly onset tachycardia, fever, and tachypnea; the patient was transferred to the intensive care unit. Urine and blood bacterial cultures and other laboratory tests excluded the possibility of the skin lesions having an infectious background. Upon further deterioration of the patient’s condition, in cooperation with the dermatology department, corticosteroids treatment was administered and the antibiotics treatment was ceased, leading to a global improvement and the patient’s discharge on the twenty-third day post-CS. The causative agent was never confirmed; however, cefazoline and metronidazole were both administered during the CS and were described as possible causative agents.

The second case report describes a presentation of TEN in the third week post-CS after antibiotics treatment for suspected endometritis [8]. On the first day of the administration of cefotaxime, the patient developed a generalized erythematous exanthema and a pulmonary embolism and she was transferred to the intensive care unit. The diagnosis was suspected toxic shock syndrome and a standard treatment of intravenous amoxicillin with clavulonic acid and methylprednisolone was administered, which led to a rapid deterioration. As the skin lesions progressed to hemorrhagic blisters, hence a TEN diagnosis was suspected and the patient was transferred to a burns intensive care unit, where, under specialist care, her condition gradually improved.

The differential diagnosis of TEN occurring during the postpartum period should include other skin conditions, especially erythema multiforme (EM), an acute skin condition involving a type IV hypersensitivity reaction, which is predominantly associated with infections and medications [9]. A patient who developed extensive skin lesions during the first postoperative day after CS was described in a case report [10]. A 27-year-old woman developed erythematous and vesicular lesions located on the abdomen and upper thighs within 24 hours of CS. The spreading of the lesions into the chest, back, and face occurred during the next day and treatment with antihistamines, metyloprednisolone, and cyclosporine A was initiated. A diagnosis of EM was confirmed histopathologically. After a full recovery, patch tests revealed a contact allergy to a disinfectant used during the CS and for colophonium and formaldehyde, which are used in adhesives and glues/surface coatings, and one of those was considered to be the causative agent.

### TEN/SJS During Pregnancy

TEN and SJS diagnosis during pregnancy have been confirmed with higher frequency than during the postpartum period. The largest single-center study on the pregnant population with a TEN and SJS diagnosis revealed that all participants, 22 women, were Human Immunodeficiency Virus (HIV) infected. In 95% of the cases, it was established that nevirapine, an antiretroviral treatment, was the culprit drug [11]. All of the pregnant patients survived the TEN/SJS treatment; however, two fetal deaths occurred during the 21st and 31st weeks of gestation, respectively. It was concluded that TEN, rather than SJS, was associated with poorer fetal outcomes; however, maternal TEN/SJS did not seem to commonly manifest in the fetus. Additionally, the authors underlined that TEN/SJS mortality rates are not increased in the HIV-positive pregnant population.

It is necessary to highlight that pregnancy may increase the risk of an SJS diagnosis by 14 times in HIV-positive women on nevirapine antiretroviral treatment [12]. The authors of the matched case–control study recommend extensive patient counseling to enable patient’s informed consent and initiation effective contraception methods upon starting the nevirapine treatment.

None of the two patients who developed TEN during the postpartum period were HIV-positive. The patient presented in our case report was also tested for HIV, in the first trimester of pregnancy and upon admission to hospital prior to CS, and remained HIV-negative.

A systematic review of the literature conducted by Sharma et al. analyzed SJS and TEN incidence during the pregnancy with a risk factors and outcomes evaluation [13]. The review included 177 pregnant patients at an average maternal age of 29.9 years old and gestational age of 24.9 weeks. It is key to highlight that HIV-positive pregnant women comprised 85% of all patients included in the review. The time from the culprit drug initiation to the reaction was reported as 27.5 days, with 90% of the causative medications being antiretroviral drugs. Only 3% of causative drugs were antibiotics and 2% were gestational drugs. The survival rate of pregnant patients was 98% and the survival rate of the neonates was 96%. The treatment methods included withdrawal of the culprit agent, supportive care and, in some cases, antibiotics, steroids, and IVIG. Preterm labor, vaginal stenosis, and vaginal adhesions were described as complications in this group. The authors concluded that SJS and TEN presentation during the pregnancy is common in young patients undergoing antiretroviral therapies, primarily nevirapine, and the mortality rates are similar to mortality rates among non-pregnant younger adults.

## 4. Discussion

The cesarean section (CS) is one of the most common surgical procedures performed globally and its frequency is constantly rising [14,15]. Importantly, CS, like all surgical procedures, has a moderate risk of infectious complications, including endometritis, CS operative site infections, and urinary tract infections. The highest risk of infectious complications is documented for emergency CS over elective CS and vaginal deliveries [16,17]. Taking into consideration the relatively high risk rate of post CS infections, a standard protocol of antibiotic treatment has been established intraoperatively and postoperatively following a suspected infection [17]. Additionally, some pregnancies are complicated by preterm rupture of the amniotic membranes, which also requires an antibiotics protocol to minimize the chances of intrauterine infections and their systemic spread [18].

Although antibacterial prophylaxis and analgesia administration are now routine with a good tolerance, it is vital to take into the consideration the possibility of adverse effects of polypharmacy in every postpartum period patient. Disorders such as TEN or SJS occur rarely. To the best of our knowledge, this case report is the second described case of TEN/SJS during the early postpartum period following a cesarean delivery [7] and the third case reported in the postpartum population [8].

No evidence was found regarding the occurrence of TEN/SJS due to perinatal antibiotics prophylaxis for group B Streptococcal colonization, or due to analgesia used during and after labor. It would be key to establish if the frequency of TEN/SJS is increased in the postpartum period population versus the general population; however, with only three cases of TEN/SJS reported during the postpartum period, this is currently not possible.

TEN and SJS are challenging conditions to diagnose and treat in the early postpartum period. Statistically, sepsis, septic shock syndrome, bacterial infections, and anaphylaxis occur more commonly and may present with similar skin lesions, so timely differential diagnosis is extremely difficult. It is possible that due to the overlapping symptoms, TEN/SJS may be underreported or misdiagnosed as septic shock syndrome or sepsis, as in the presented cases [7,8]. Differential diagnosis of TEN during the postpartum remains extremely difficult, as the generalized symptoms of hyperpyrexia, tachycardia, and tachypnea, upon rapid exacerbation in the postpartum period, will undoubtedly point clinicians in the direction of a sepsis/septic shock diagnosis. The skin lesions in the initial stages may also be mistaken for an allergic reaction to antibiotics or operative field disinfectants [18,19].

In the described case report, a rapid improvement in the patient’s condition was observed soon after IVIG administration; this is considered to be an experimental method of treatment, which has been recommended in previously published papers [20,21,22]. Through this case, we would like to highlight the importance of a multidisciplinary approach and cooperation in the diagnosis and treatment of patients with rare and alarming symptoms, which many practitioners may have never been seen before during their clinical practice. It is crucial to keep in mind the possibility of a surprising diagnosis such as TEN or SJS, especially in cases in which multiple analgesia and antibiotics are administered to young and previously healthy patients during pregnancy and in the postpartum period, whose symptoms are exacerbated after a standard sepsis treatment.

Early management and multidisciplinary treatment are necessary to maximize patients’ survival rates. The treatment of patients with TEN/SJS should be initiated with an early-culprit drug withdrawal. TEN/SJS patients, like burns patients, require intensive care with adequate fluid resuscitation, respiratory support, pain control, infection prophylaxis, anticoagulant therapy, nutritional treatment, and gastric ulcer prophylaxis. The key to local treatment of patients with TEN/SJS is the use of non-adherent dressings that do not damage the epidermis during wound-dressing changes. The aim of systemic treatment is the purification of the bloodstream from the causative agent. Some studies suggest that the most efficient way to clarify the serum of TEN/SJS patients is through a combination of IVIG and plasmapheresis; however, there is no solid consensus on the treatment [23,24,25]. Immunomodulatory therapy can reduce mortality five times in comparison to that of patients with immunosuppression or cases which lack a full protocol [26]. Based on our clinical experience, IVIG treatment of TEN/SJS during the postpartum period could be considered upon ineffective culprit drug withdrawal and steroid therapy.

## 5. Conclusions

TEN and SJS are rare, severe systemic reactions which are predominately attributed to exposure to medications which may occur more often during the postpartum period due to polypharmacy and postpartum pro-inflammatory immunomodulation. Early diagnosis and treatment initiated with immediate cessation of the causative agent and administration of a full protocol of immunomodulatory therapy can result in a timely recovery. Postpartum period patients, particularly those after a cesarean section, require carefully tailored management, as the post-delivery period is characterized by complex hormonal fluctuations. A sudden decline in estrogen and progesterone levels leads to a significant alteration in immune homeostasis, shifting the system toward a pro-inflammatory state that persists for several postpartum weeks [27,28]. Although the pathogenesis of TEN/SJS is not yet fully understood, immunological disregulation is considered a key factor, as exemplified by the markedly increased risk of TEN/SJS in immunocompromised individuals, including patients with HIV. This clearly supports the central role of the immune system in disease development.

In the present case, both postpartum homeostatic changes, along with physiological stress and the systemic impact of a cesarean section, likely contributed to the immune imbalance. One plausible hypothesis linking the postpartum period with TEN/SJS is the estrogen [29] and progesterone-mediated modulation of Major Histocompatibility Complex (MHC) class I expression shown in in vitro models [30]. Both estrogen and progesterone are physiologically increased during pregnancy and subsequently up-regulated following the sharp decline during the postpartum period, which may potentially enhance MHC class I expression. In this context, concepts proposed in TEN/SJS pathogenesis emphasize the role of MHC class I-mediated immune activation. An increased availability of these receptors may enhance the likelihood of aberrant drug-specific immune responses. Because MHC class I plays a central role in the immunopathogenesis of TEN, its up-regulation may facilitate a cytotoxic T-cell activation either through a direct, non-covalent interaction of the drug with the MHC class I complex or the T-cell receptor, or by altering the repertoire of peptides presented, leading to a misrecognition of the self-antigens.

In conclusion, a multidisciplinary approach and the cooperation of dermatologists and obstetricians are crucial for success in the therapy of women diagnosed with TEN and SJS during the fragile postpartum period.

## Figures and Tables

**Table 1:** Analysis of patient’s condition, skin lesion changes, laboratory results, and medical interventions during the differential diagnosis and treatment process.

Timeline (Days)	1.	2.	3.	4.	5.	6.	7.	8.	9.	10.	11.	12.	13.	14.	15.
Medical Intervention ** ** and ** ** differential diagnosis	Admission to Obstetrics Department	Cesarean section (CS) due to fetal distress	1st day post CS	2nd day post CS Cervical swab result: Ureaplasma urealiticum	3rd day post CS Suspicion of a mild allergic reaction to Ampicillin	4th day post CS Dermatology and allergology consultation	5th day post CS Suspicion of sepsis Bacterial blood cultures Broad-spectrum antibiotics	6th day post CS Dermatology & Microbiology consultation Suspicion of TEN/SJS/SSSS	6th day post CS Dermatology consultation Confirmation of TEN	7th day post CS Dermatology and ophthalmology consultation 1st day of IVIG	8th day post CS 2nd day of IVIG	9th day post CS 3rd day of IVIG	10th day post CS Dermatology consultation	11th day post CS	12th day post CS Qualification for discharge
Patient’s condition	Stable, Preterm rupture of membranes	Stable	Stable	Stable	Initial skin reaction: 20 min after Ampicillin i.v.	Exacerbation of skin lesions	Further exacerbation of skin lesions, febrile, weakness	Further exacerbation of skin lesions	Further exacerbation of skin lesions	Stabilization of skin lesions	Stabilization of skin lesions General improvement	Stabilization of skin lesions General improvement	Stable	Stable	Stable
Location of skin lesions	-	-	-	-	Chest, abdomen	Chest, abdomen	Chest, abdomen, upper and lower extremities	Chest, abdomen, upper and lower extremities, face	Chest, abdomen, upper and lower extremities, face	Chest, abdomen, upper and lower extremities, face	Chest, abdomen, upper and lower extremities, face	No new lesions, epithelization	No new lesions, epithelization	No new lesions, epithelization	No new lesions, epithelization
Character of skin lesions	-	-	-	-	Maculopapular rash	Maculopapular rash	Maculopapular rash Petechiae	Maculopapular rash Petechiae	Fluid-filled blisters Maculopapular rash Petechiae	Fluid-filled blisters Maculopapular rash Petechiae	Fluid-filled blisters Maculopapular rash Petechiae	Healing of fluid-filled blisters epithelization	Healing of fluid-filled blisters epithelization	Healing of fluid-filled blisters epithelization	Predominantly healed fluid-filled blisters epithelization
Pharmacotherapy	Oxytocin	Ampicillin Ceftriaxone Spinal anesthesia Ibuprofen Paracetamol	Ampicillin Paracetamol Ibuprofen Enoxaparin	Ampicillin Azithromycin Ibuprofen Paracetamol Enoxaparin	Ampicillin cessation Azithromycin Ibuprofen Paracetamol Cetirizine Enoxaparin	Azithromycin Ibuprofen cessation Paracetamol Cetirizine Cutivate ointment Enoxaparin	Azithromycin Paracetamol Cetirizine Cutivate ointment Prednisone Clemastine Hydrocortisone Enoxaparin Clindamycin Metronidazole	Azithromycin Paracetamol Cetirizine Cutivate ointment Prednisone Clemastine Hydrocortisone Enoxaparin Clindamycin Metronidazole	Paracetamol Cetirizine Cutivate ointment Prednisone Clemastine Hydrocortisone Enoxaparin Clindamycin Metronidazole	Paracetamol Cetirizine Cutivate ointment Prednisone Clemastine Hydrocortisone Enoxaparin Clindamycin Metronidazole	Paracetamol Cetirizine Cutivate ointment Prednisone Clemastine Hydrocortisone Enoxaparin Clindamycin Metronidazole	Paracetamol Cetirizine Cutivate ointment Prednisone Clemastine Hydrocortisone Enoxaparin Clindamycin Metronidazole	Paracetamol Cutivate ointment Prednisone Enoxaparin Clindamycin Metronidazole Methyldopa	Paracetamol Cutivate ointment Prednisone Enoxaparin Methyldopa	Paracetamol Cutivate ointment Prednisone Enoxaparin Methyldopa
Vital signs Blood pressure (BP mm/Hg) Heart rate (HR) Oxygen Saturation (Sat. O_2_%) Body Temperature (Temp. °C)	BP 130/80 HR 90 Sat. O_2_ 100% Temp. 36.8 °C	BP 120/70 HR 80 Sat. O_2_ 100% Temp. 36.7 °C	BP 115/77 HR 85 Sat. O_2_ 100% Temp. 36.8 °C	BP 120/70 HR 80 Sat. O_2_ 100% Temp. 36.7 °C	BP 110/70 HR 92 Sat. O_2_ 100% Temp. 36.8 °C	BP 115/72 HR 90 Sat. O_2_ 100% Temp. 36.9 °C	BP 99/60 HR 130 Sat. O_2_ 99% Temp. 40 °C	BP 100/63 HR 120 Sat. O_2_ 99% Temp. 38.8 °C	BP 110/60 HR 110 Sat. O_2_ 99% Temp. 38.0 °C	BP 112/65 HR 100 Sat. O_2_ 99% Temp. 37.1 °C	BP 110/59 HR 101 Sat. O_2_ 99% Temp. 37.2 °C	BP 119/60 HR 98 Sat. O_2_ 99% Temp. 37.0 °C	BP 130/83 HR 76 Sat. O_2_ 100% Temp. 36.8 °C	BP 122/70 HR 70 Sat. O_2_ 100% Temp. 36.6 °C	BP 110/65 HR 64 Sat. O_2_ 100% Temp. 36.6 °C
Hemoglobin [g/dL] ** ** Normal value ** ** 12–15	12.4	11.9	11.9	-	-	-	12.2	12.8	10.2	10.8	-	8.9	-	-	10.8
White blood cells [×10^9^/L] Normal value 4–10	11.97	16.67	16.68	-	-	-	17.68	19.32	12.38	10.03	-	9.43	-	-	8.22
C-reactive protein [mg/L] Normal value 0–5	21	22	26	18	24	-	106 130	182	223	126	-	44	18	-	-
Procalcitonin ** ** Normal value ** ** 0.00–0.5	-	-	-	-	-	-	0.12	0.4	-	-	-	-	-	-	-
D-Dimer [ug/L] ** ** Normal value ** ** <500	-	-	-	-	-	-	12,230	35,900	17,524	-	-	3281	-	-	-
Creatinine [mg/dL} ** ** Normal value ** ** 0.55–1.02	-	-	-	-	-	-	0.96	1.07	0.85	0.77	-	-	-	-	-
LDH [U/L] ** ** Normal value ** ** 125–220	-	-	-	-	-	450	270	-	-	-	-	-	-	-	-

**Table 2:** Differential diagnosis with evaluation of the supporting and excluding clinical findings.

Suspected Diagnosis	Findings Supporting the Diagnosis	Findings Against the Diagnosis
Stevens–Johnson Syndrome/Toxic Epidermal Necrolysis (SJS/TEN)	Skin involvement of up to 10% of the BSA with epidermal detachment (positive Nikolsky sign) and up to 90% of the BSA with blistering Oral and genital mucosal erosions; conjunctival changes Close temporal association with drug exposure	Final diagnosis
Drug-induced exanthema (morbilliform eruption)	Initially morbilliform drug skin lesions involving the trunk and extremities Close temporal association with drug exposure Early intense pruritus	Blistering, mucosal erosions, and epidermal detachment
Acute Generalized Exanthematous Pustulosis (AGEP)	Presence of numerous small pustules on the trunk and extremities in the early phase	Prominent mucosal involvement Significant blistering is rarely seen in AGEP
Staphylococcal Scalded Skin Syndrome (SSSS)	Early systemic features of inflammation, skin fragility and blistering	Mucosal erosions present (oral, genital, ocular)—not a feature of SSSS Drug trigger was more likely than *Staphylococcus aureus* toxin
Mycoplasma-Induced Rash and Mucositis (MIRM)	Presence of oral and genital mucosal erosions	Extensive skin involvement, whereas MIRM typically presents with limited cutaneous disease Negative IgM and IgG for Mycoplasma pneumoniae No radiographic evidence of pneumonia
Postpartum Sepsis	Tachycardia, hypotension, hyperpyrexia Elevation of C-reactive protein and white blood cell count	Negative blood tests for bacterial cultures Procalcitonin level was within the normal range Skin lesions not typical of sepsis

**Table 3:** ALDEN scoring was performed to determine the likelihood of drug-induced causation of the skin lesions.

Suspected Drug	Delay from Initial Drug Component Intake to Onset of Reaction	Drug Present in the Body on Index Day	Prechallenge/Rechallenge	Dechallenge	Type of Drug	Other Cause	ALDEN Score	Causal Link
Ampicillin	Likely +1	Definite 0	Not done/unknown 0	Neutral 0	Associated +2	N/A	3	Possible
Azithromycin	Likely +1	Definite 0	Not done/unknown 0	Negative −2	Strongly associated +3	N/A	2	Possible
Ceftriaxone	Likely +1	Doubtful −1	Not done/unknown 0	Neutral 0	Strongly associated 3+	N/A	3	Possible
Enoxaparin	Likely +1	Definite 0	Not done/unknown 0	Negative −2	Not suspected −1	N/A	−2	Very unlikely
Ibuprofen	Likely +1	Definite 0	Negative −2	Neutral 0	Strongly associated +3	N/A	2	Possible
Oxytocin	Likely +1	Excluded −3	Not done/unknown 0	Neutral 0	Not suspected −1	N/A	−3	Very unlikely
Paracetamol	Likely +1	Definite 0	Negative −2	Negative −2	Associated +2	N/A	−1	Very Unlikely

**Table 4:** SCORTEN and ABCD-10 scores were performed to determine the estimated in-hospital mortality rate due to SJS/TEN diagnosis.

SCORTEN		ABCD-10	
Feature	Score	Feature	Score
Age ≥ 40 years	0	Age ≥ 50 years	0
Serum bicarbonate level (<20 mmol/L)	0	Serum bicarbonate level (<20 mmol/L)	0
Cancer or hematologic malignancy	0	Active cancer	0
-	-	Dialysis prior to admission	0
BSA > 10%	0	BSA > 10%	0
Heart rate ≥ 120 bpm	0	-	-
Serum urea level (>10 mmol/L)	0	-	-
Serum glucose level (>14 mmol/L)	0	-	-
Total score	0–estimated mortality rate–3.2%	Total score	0–estimated mortality rate–2.3%

## Data Availability

No new data were created or analyzed in this study.

## Abbreviations

AGEP	Acute Generalized Exanthematous Pustulosis
CRP	C-Reactive Protein
CS	Cesarean Section
CTG	Cardiotocography
EM	Erythema Multiforme
GBS	Group-B Streptococcus.
HIV	Human Immunodeficiency Virus
IVIG	Intravenous Immunoglobulins
MHC	Major Histocompatibility Complex
MIRM	Mycoplasma-Induced Rash and Mucositis
NSAIDs	Non-Steroidal Anti-Inflammatory Drugs
SJS	Stevens–Johnson Syndrome
SSSS	Staphylococcal Scalded Skin Syndrome
BSA	Total Body Surface Area
TEN	Toxic Epidermal Necrolysis
